# Design of Alginate-Based Bionanocomposites with Electrical Conductivity for Active Food Packaging

**DOI:** 10.3390/ijms22189943

**Published:** 2021-09-14

**Authors:** Zélia Alves, Nuno M. Ferreira, Sónia Mendo, Paula Ferreira, Cláudia Nunes

**Affiliations:** 1 Department of Chemistry, CICECO-Aveiro Institute of Materials, University of Aveiro, 3810-193 Aveiro, Portugal; zeliaralves@ua.pt; 2 Department of Materials and Ceramic Engineering, CICECO-Aveiro Institute of Materials, University of Aveiro, 3810-193 Aveiro, Portugal; 3 Department of Physics, i3N, University of Aveiro, 3810-193 Aveiro, Portugal; nmferreira@ua.pt; 4 Department of Biology, CESAM, University of Aveiro, 3810-193 Aveiro, Portugal; smendo@ua.pt

**Keywords:** alginate, reduced graphene oxide, zinc oxide, antioxidant, antimicrobial

## Abstract

Bionanocomposite materials have been designed as a promising route to enhance biopolymer properties, especially for food packaging application. The present study reports the preparation of bionanocomposite films of alginate with different loadings of pure reduced graphene oxide (rGO) or of mixed zinc oxide-rGO (ZnO-rGO) fillers by solvent casting. Sepiolite is used to make compatible rGO with the hydrophilic matrix. The addition of fillers to alginate matrix maintains the low water solubility promoted by the calcium chloride treatment, and, additionally, they demonstrate a weaker mechanical properties, and a slight increase in water vapor permeability and wettability. Due to the properties of ZnO-rGO, the alginate bionanocomposites show an increase of electrical conductivity with the increase of filler content. While the highest electrical conductivity (0.1 S/m) is achieved by the in-plane measurement, it is in the through-plane measurement the remarkable enhancement of almost 30 times greater than the alginate film. With 50% of ZnO-rGO filler, the bionanocomposites present the highest antioxidant and antibacterial activities. The combination of electrical conductivity with bioactive properties makes these films promising not only to extend food shelf-life but also to allow packaged food sterilization at low temperature.

## 1. Introduction

The development of composite materials for the active food packaging field has recently been explored to answer the consumers’ concerns with the safety and quality of food products, the health, and the environment. Besides the handling and storage conditions, an active food packaging material can enable to preserve the food product, resulting in the extension of its shelf-life [1]. Therefore, following the trend of an eco-friendly food packaging material with active functionalities, the recent research has driven efforts to produce bio-based plastics targeting the simultaneous use of biopolymers and active agents [2]. Biopolymers, including polysaccharides, proteins, and lipids, have been regarded as more sustainable materials because they are natural, came from renewable resources and may be biodegradable and compostable, satisfying the current environmental challenges by solving the waste disposal problems to some extent. Moreover, they can be chemically functionalized and blended with other biodegradable polymers, plasticizers, compatibilizers, and/or active fillers to improve their performance, being good alternatives the petroleum-based polymers in a diversity of applications, from food packaging to biomedical fields [3,4,5].

Among the natural biopolymers, the polysaccharides have been explored as alternative materials to replace the non-environmental polymers due to their biodegradability, high availability, and low cost. Alginate is a linear anionic polysaccharide, mainly extracted from brown seaweed, composed by two different sugars residues, mannuronic and guluronic acid, linked by (1– 4) glycosidic bond [6]. Besides its application in the food industry due to gelling properties in presence of bivalent cations (Ca^2+^) [7], alginate presents a good film-forming capability to be explored as coatings, edible films, and/or food packaging materials [8,9]. Alginate films have good characteristics for a food packaging material such as transparency, biodegradability, biocompatibility, and non-toxicity [8]. However, their highly solubility in water, poor mechanical and gas barrier properties represent some limitations. In line to overcome these issues of alginate films and make them suitable as a food packaging material, alginate biopolymer can be combined with functional agents. In fact, the development of biocomposite films based on biopolymers with nanomaterials and/or biologically active ingredients has been extensively explored to improve the properties of these biopolymers and to impart additional functionalities [10,11,12]. Addressing the preparation of active food packaging systems, alginate has also been on studied as a matrix to formulate biocomposites [13,14]. Among the fillers employed, the effect of cellulose [15], plant extracts [16], clays [17], metal oxides (e.g., ZnO [18], SiO_2_ [19], TiO_2_ [20]), and carbon-based materials (e.g., graphene oxide (GO) [21,22], rGO [23], carbon nanotubes [24]) have been studied on alginate matrix. The incorporation of rGO and ZnO nanoparticles as fillers has improved the mechanical and barrier performance of films [19,25,26], beyond to impart active properties, including antimicrobial activity [27], antioxidant activity [26], and UV light barrier [26]. The combination of these two materials as a multifunctional filler (ZnO-rGO) reveal that their intrinsic properties are maintained or improved by synergistic effects [28,29]. Another appealing functional property of ZnO-rGO composite is the imparted electrical conductivity to insulating biopolymers [27]. Although there are good properties, the toxic effect of these nanomaterials on living cells and organs should not be neglected. Recently, the biocompatibility and toxicity of rGO and ZnO in vivo and in vitro have been evaluated, and the studies demonstrated that the potential toxic effect varies according to the dose and rote of administration, and of the method of synthesis and physicochemical properties of the nanomaterials [30,31,32,33] However, as these nanomaterials are incorporated into the biopolymer matrix, the studies of migration from the packaging material to the food products have been done and claimed that there is no migration or that migration is below the upper limit considered safe by government entities [34].

A food-grade packaging material that allows the electrical current to pass through it will permit the sterilization of food products through technologies such as pulsed electric field [35] and ohmic heating [36]. As the foods’ microbial inactivation occurs inside the packaging material, the post-process contamination that compromises the food safety after the treatment is avoid. Thus, the addition of electrically and active ZnO-rGO filler to alginate matrix is an interesting approach to produce a bionanocomposite packaging material suitable to protect and preserve the food products. Although the dispersion of electrically conductive carbon-based materials into an aqueous polymeric matrix is weak [37,38], ZnO-rGO can be exfoliated and intercalated under sonication with sepiolite fibres. This natural fibrous silicate clay has been reported to improve the mechanical and barrier properties of biopolymers, but also to stabilize carbon-derivatives in aqueous solutions [39,40]. 

In this context, this work aimed to develop alginate-based films with the incorporation of different loadings of the multifunctional ZnO-rGO/sepiolite composite for a food packaging application. Antimicrobial and antioxidant activities have been widely explored in the last decade to produce active food packaging materials; however, to the best of our knowledge, the combination of these active properties with the electrical conductivity is studied here for the first time. The alginate-based films were investigated in terms of structure, morphology, solubility, wettability, water vapour permeability, and mechanical performance. In addition, the antioxidant capacity, antimicrobial activity, and electrical conductivity was determined to assess their potential application as an electrical and active food packaging material to increase the safety and extend the shelf-life of food products.

## 2. Results and Discussion

**Figure 1:**
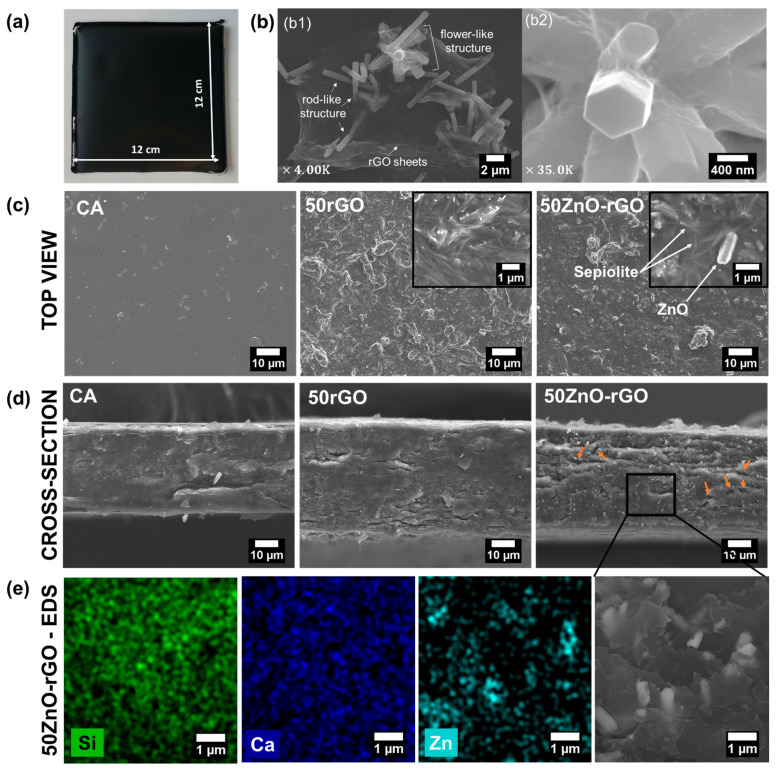
Photography of an alginate-based film with the incorporation of rGO or ZnO-rGO dispersed in sepiolite (**a**). SEM micrograph of ZnO-rGO filler (**b1**) and a magnification of flower-like ZnO particles attached/wrapped on the rGO sheets (**b2**). SEM micrographs of CA, 50rGO, and 50ZnO-rGO films: top view, with magnification displayed in the upper right corner of the respective image (**c**), and cross-section (**d**). A cross-section magnification image of 50ZnO-rGO film and corresponding EDS elemental maps (**e**). Orange arrows indicate empty cavities in the film; white short arrows highlight the rod-like structure and the rGO sheets; white long arrows highlight the sepiolite and ZnO particles.

Bionanocomposite films were prepared by solvent casting from mixtures of aqueous solutions of alginate with rGO or ZnO-rGO previously dispersed into the sepiolite fibres. The transparent alginate film turns black and macroscopically homogeneous with the incorporation of both fillers, indicating the good dispersion of filler in the biopolymer matrix (Figure 1a). Different amounts of filler were incorporated into the alginate matrix, ranged from 25 to 50% of the alginate weight, which consequently allows the films thickness to increase from 32.3 µm to between 56.1 and 86.6 µm. The film control of alginate (CA) and the bionanocomposites films with rGO (25rGO, 30rGO, 40rGO, and 50rGO) and with ZnO-rGO (25ZnO-rGO, 30ZnO-rGO, 40ZnO-rGO, and 50ZnO-rGO) were characterized in terms of their structure, morphology, water solubility, wettability, WVP, and mechanical, antioxidant, antimicrobial, and electrical properties. 

### 2.1. Morphology and Structure of Fillers and Alginate-Based Films

The most used route to synthesize the ZnO-rGO filler, considering the food contact application, is the in situ hydrothermal method, where mild temperatures and pressures are involved. This green and one-pot methodology is used to grow high crystalline ZnO particles and, at the same time, reduce the GO. The SEM images of ZnO-rGO filler (Figure 1(b1)) shows that ZnO particles have a length of 2.95 ± 1.31 µm and a width of 634.01 ± 121.49 nm and present a rod- and flower-like shape. Moreover, the ZnO particles are distributed and immersed in the thick layers of rGO, and from Figure 1(b2), we also observed wrinkles and ripples on the surface of ZnO particles, suggesting the occurrence of few layered rGO sheets on their surface. These observations indicate that ZnO is attached and/or wrapped to the rGO sheets and this interaction allows a good dispersion of ZnO particles. The SEM micrographs of the top view and cross-section of the CA, 50rGO, and 50ZnO-rGO films are shown in Figure 1c–e. The neat alginate film exhibits a smooth surface, but the incorporation of the fillers enhances the roughness of the surface. Recently, the literature demonstrated that the complexation of alginate chains with Ca^2+^ and the fillers addition increase the irregularities and roughness of the alginate films’ surface, besides to show a more disordered structure [41,42]. From the inset magnification image of 50ZnO-rGO, it is clearly visible that the sepiolite fibres and the ZnO particles are present on the film surface (Figure 1c). Concerning the cross-sectional view (Figure 1d), the incorporation of the fillers turns films thicker and produces a more stratified internal structure due to the self-assembly of rGO sheets during the solvent casting process. Through cross-section image of 50ZnO-rGO film, it is possible to verify that ZnO particles are homogeneously dispersed and randomly oriented into the alginate matrix. The elemental analysis of 50ZnO-rGO film by EDS (Figure 1e) confirms the good distribution of Zn, but also the Si and Ca, that are strongly present due to the sepiolite used to disperse the particles and the CaCl_2_ solution used to complex with alginate, respectively. The SEM images confirm that the synthesis of ZnO particles together with the reduction of GO, and subsequent dispersion in the sepiolite fibres ensure the good compatibility with the alginate matrix. 

**Figure 2:**
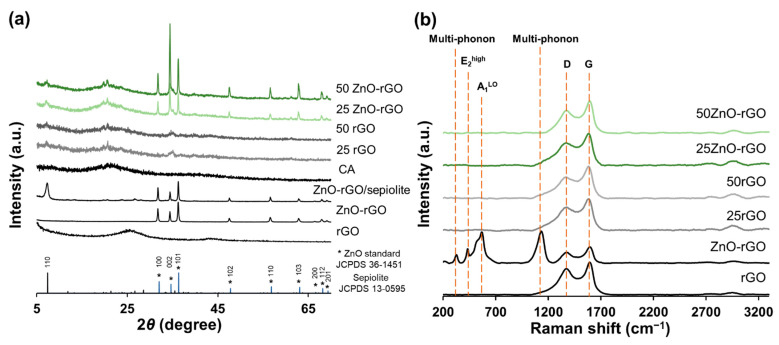
(**a**) XRD diffraction patterns and (**b**) Raman spectra of fillers (rGO and ZnO-rGO) and alginate-based films with the incorporation of 25 and 50% of filler. (*) asterisk indicates the wurtzite crystalline phases of ZnO standard (JCPDS 36-1451).

The XRD patterns of rGO, ZnO-rGO, and ZnO-rGO/sepiolite are represented in Figure 2a. The reflections of rGO, related with the disordered of graphite crystallites formed during the reduction reaction, appeared at the diffraction angles 2*θ* of 25.4° and 42.9°, corresponding to the (002) and (100) planes, respectively [43]. The XRD diffractogram of synthetized ZnO-rGO composite shows the characteristic diffraction peaks of hexagonal ZnO (JCPDS card no. 361451), which correspond to the wurtzite structure. This suggests the successful formation of hexagonal ZnO particles in simultaneous to the reduction of GO during the hydrothermal process. However, peaks corresponding to the rGO are not observed in the multicomponent filler and this could happen due to the high crystallinity of ZnO particles present on the surface of rGO or due to the stacking disorder of rGO sheets in the presence of ZnO particles, as observed in other studies [44,45]. The XRD pattern of the ZnO-rGO filler in sepiolite shows the presence of the zinc oxide peaks, indicating that the crystal structure of the ZnO is preserved. Moreover, sepiolite reflections are also present, as the most intense peak observed at 2*θ =* 7.3°, which corresponds to the (110) reflection of the silicate [39]. The crystallography patterns of the lowest (25rGO and 25ZnOrGO) and the highest (50rGO and 50ZnOrGO) content of filler added to alginate film are also represented in Figure 2a. The bionanocomposite films show the peaks related with the incorporation of rGO or ZnO-rGO dispersed in sepiolite, revealing the preservation of the filler structure. In addition, one broad peak is possible to observe at 2*θ* = 21.3° which is related to the alginate biopolymer [46]. The crystallographic peaks related to ZnO phase display increasing intensity as ZnO content increases. 

Raman spectra of the rGO, ZnO-rGO powders and the alginate films obtained with these powders in the percentage of 25 and 50% are presented in Figure 2b. The rGO and ZnO-rGO powders show the typical bands that characterize the graphene-like materials, the D and G band, which lie at around 1368 and 1592 cm^−1^, respectively [47]. The D band occurs as a result of sp^3^ carbons present in the lattice introduced after the oxidation process of GO and persists after the reduction of GO. Thus, the D band intensity gives an indication of structural defects or disorder degree within the material. The G band is related to the in-plane sp^2^ aromatic domains of the graphite carbon structure, representing the crystallinity of the material [48]. The ZnO-rGO filler also shows some bands at low wavelengths (250–1200 cm^−1^) that are typical of ZnO particles, revealing that these particles are well incorporated into the rGO sheets. The spectrum shows two basic phonon modes of the hexagonal wurtzite phase ZnO at 434 and 567 cm^−1^, which represents the E_2_^H^ and A_1_^LO^, respectively. The E_2_^H^ is associated with the vibration of oxygen atoms and can be related to the degree of crystallization whereas A_1_^LO^ band is associated with the defects such as oxygen deficiencies, zinc interstitial, or their complexes [49,50]. The band at 327 cm^−1^ and 1134 cm^−1^ represent the multi-phonon scattering processes of ZnO [51]. Concerning the Raman spectra of alginate-based films, all samples show the characteristic D and G bands, representing the presence of rGO into the biopolymer matrix. Moreover, both bands have similar frequencies than the ones observed for the rGO filler, demonstrating that rGO structure was preserved during the film formation. The characteristics peaks related to the ZnO particles are absent in the Raman spectrum of the films were ZnO-rGO filler was incorporated. As ZnO particles are well impregnated into the solidified alginate polymer, it is difficult to detect their presence in Raman spectroscopy, which is consistent with other studies [52]. The Raman films’ characterization in complementarity with the XRD characterization allows us to conclude that ZnO-rGO filler integrity is preserved as a whole after incorporation onto the alginate matrix, ensuring that its active properties and electrical conductivity are imparted to alginate film.

### 2.2. Solubility, Wettability, and Water Vapour Permeability of Films

**Figure 3:**
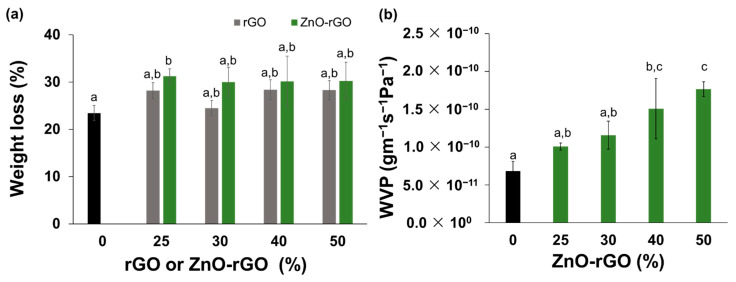
(**a**) Water solubility (weight loss (%)) of CA and bionanocomposite films (25rGO, 25ZnO-rGO, 30rGO, 30ZnO-rGO, 40rGO, 40ZnO-rGO, 50rGO, and 50ZnO-rGO). (**b**) Water vapor permeability (WVP) of CA and bionaocomposite films with ZnO-rGO filler. Different letters represent significant (*p* < 0.05) values (*n* = 3).

One of the major problems of alginate-based films to be applied as a food packaging material is their completely dissolution upon contact with water, particularly because food has high-water content. To avoid this drawback all the films were immersed in aqueous CaCl_2_ solution to promote a crosslinking of alginate by complexation. In this way, Ca^2+^ ions will migrate into the alginate network, stabilizing the polysaccharide conformation via ionic interactions. An “egg-box” structure is formed due to the interactions of Ca^2+^ ions with carboxylic groups of guluronic monomers, forming a water-insoluble network that allows to increase the water resistance of alginate-based films [53]. In this study, a solubility test obtained after immersion of the alginate-based films for seven days in distilled water (pH 6) was performed (Figure 3a). The weight loss of CA film and bionanocomposite films with rGO or ZnO-rGO fillers dispersed in sepiolite have similar values, with weight loss percentages ranged from 23 to 31%. As bionanocomposite films do not have a significantly different weight loss from the control film or from each other, regardless the filler loading, the solubility can be explained by the diffusion of glycerol molecules to the medium, as already reported in the literature [54]. In addition, all alginate-based films keep their structure intact which confirms that the crosslinking less exposes the hydrophilic sites along the alginate chains as well their availability to bind to water molecules [41]. Figure S1 shows the surface wettability of the alginate-based films determined by the sessile drop method using ultrapure water placed on the film surface. The hydrophilic and hydrophobic characteristics of the film surface can be measured through the contact angle formed between drops of ultrapure water and the film surface [55]. The contact angle values range from 54.2° to 18.4°, allowing us to infer that all samples have a hydrophilic surface. The highest value is achieved by CA film, whereas all bionanocomposites have significantly lower contact angles which is a consequence of the sepiolite clay used to disperse the rGO and ZnO-rGO fillers. As observed by other authors, films of alginate loaded with neat sepiolite show a decrease in the water contact angle due to its strong hydrophilic character, explained by the high density of silanol groups (−SiOH) [39,56]. Moreover, as it can be seen in the SEM micrographs (Figure 1c), the addition of rGO and ZnO-rGO enhances the film surface roughness, enlarging the surface area and contributing to the increase of surface wettability [57]. Indeed, the presence of remaining hydroxyl groups in the rGO structure and the carboxylic groups on the alginate film surface may establish a good interaction with water molecules, decreasing the water contact angle [27]. The water vapour permeability values measured for the CA film and the different bionanocomposites with ZnO-rGO filler are shown in Figure 3b. The CA film shows a WVP of 6.8 × 10^−11^ gm^−1^s^−1^Pa^−1^, a value in the same order of magnitude reported by other authors [58]. After the addition of ZnO-rGO composite, the WVP tends to increase with the filler loading, and it is significantly different from the CA film when the concentration of ZnO-rGO filler is above 40%. This increase of 2.6 times of the WVP value can occur due to the presence of ZnO-rGO in the alginate matrix, which leads to the formation of porous structures between the alginate chains [59]. Besides that, high concentration of ZnO-rGO hinders at some extent the crosslinking between the alginate chains and the Ca^2+^ ions, leading to a different reorganization of alginate chains and increasing their interstitial space [23]. Therefore, an easy pathway is formed helping the diffusion of water molecules through the film, which can be confirmed by the cross-section SEM image of 50ZnO-rGO where empty cavities are observable (signed with orange arrows in Figure 1d). On the other hand, the effective Ca^2+^ complexation on the CA film reduces the alginate chains mobility and, consequently, the WVP through the film, a hypothesis that is supported by other authors [60]. The addition of fillers to alginate matrix maintains the low water solubility promoted by the crosslinking with Ca^2+^ ions, and do not have a strong impact on water vapor permeability and wettability. These properties turn the alginate bionanocomposite films with potential to be used as packaging materials for fresh food products.

### 2.3. Mechanical Properties

**Figure 4:**
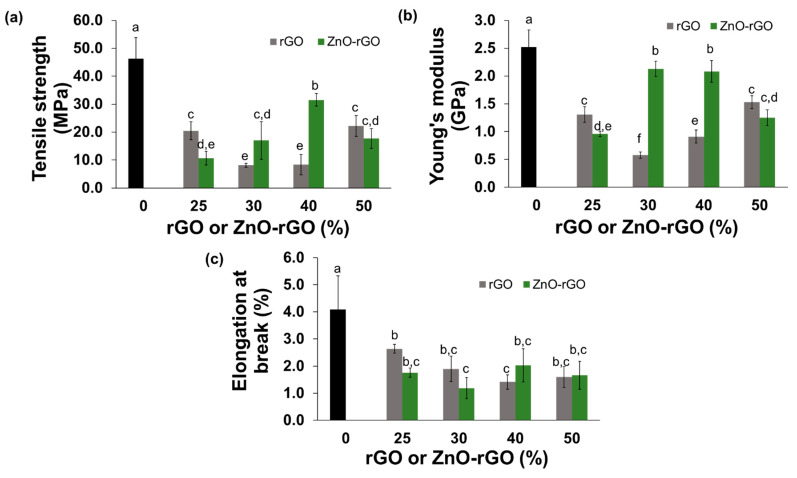
Mechanical properties: Tensile strength (**a**), Young’s modulus (**b**), and Elongation at break (**c**), of CA and bionanocomposites with different loading of rGO and ZnO-rGO. Different letters represent significant (*p* < 0.05) values (*n* = 6).

The effect of the incorporation of rGO or ZnO-rGO, previously dispersed in sepiolite, on alginate films were evaluated in terms of mechanical properties, namely tensile strength (TS), elongation at break, and Young’s modulus (Figure 4). CA film is the sample with the highest TS (46.3 MPa) whereas the incorporation of fillers in its matrix decreased this parameter. Although the 40ZnO-rGO film represents the sample with the greatest resistance (31.6 MPa) among the bionanocomposite films (Figure 4a), the observed reduction of TS compared with the CA film can happen due to the breakup of the film network caused by the fillers incorporation, in accordance with other studies [39,61]. Young’s modulus of CA film is 2.52 GPa, a value that is accordance with the literature for alginate films with 30% of glycerol immersed in a CaCl_2_ solution of 2% [62]. A slight decrease of the YM value occurred with the addition of 30 and 40% of ZnO-rGO filler, but the films with the other concentrations of ZnO-rGO filler and all the films with the rGO addition, have a more pronounced decrease of YM, about 1.6 to 4.4 times (Figure 4b), indicating that are significantly less stiffness comparing with the CA film. The significantly decrease of elongation at break value (Figure 4c) of all the bionanocomposite films compared with the CA film indicates the loss of alginate-based films flexibility with the filler’s inclusion. In general, the resistance and stiffness of bionanocomposites are decreased when compared with the CA film and this can result by a weaken interfacial interaction between the fillers and the biopolymer. The strong resistance in CA film can be explained by the formation of the “egg-box” structure which results of alginate interaction with Ca^2+^ ions. The enhancement of these ionic linkages decreases the interaction of alginate with H_2_O molecules which leads to stronger and more cohesive films and, consequently, decreases the film flexibility [41]. In contrast, the incorporation of fillers into the alginate matrix seems to obstacle the diffusion of Ca^2+^ ions and thus the degree of crosslinking is weaker and disordered, especially in the inner parts, which results in more breakable films. Despite this effect, the 40ZnO-rGO film is the bionanocomposite with the highest mechanical properties (TS: 31.6 MPa and YM:2.0 GPa) under study.

### 2.4. Electrical Properties

**Figure 5:**
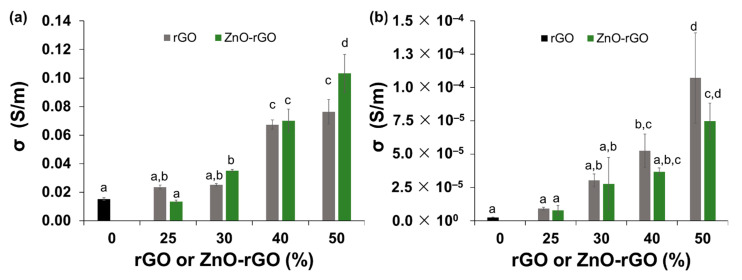
In-plane (**a**) and through-plane (**b**) electrical conductivity of alginate and bionanocomposites (25rGO, 25ZnO-rGO, 30rGO, 30ZnO-rGO, 40rGO, 40ZnO-rGO, 50rGO, and 50ZnO-rGO). Different letters represent significant (*p* < 0.05) values (*n* = 6).

**Figure 6:**
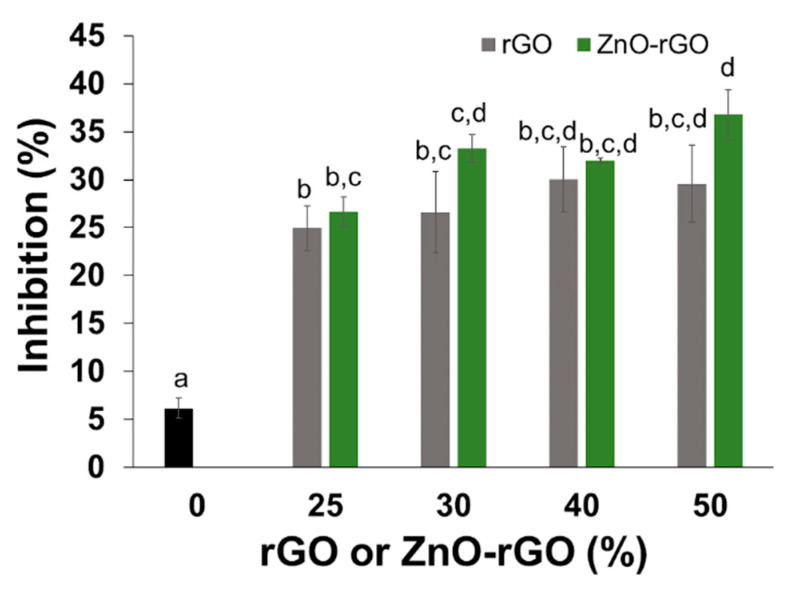
Antioxidant activity (inhibition %) of CA and rGO or ZnO-rGO bionanocomposites after 30 min of incubation in ABTS radical cation Different letters represent significant (*p* < 0.05) values (*n* = 3).

The electrical conductivity of CA and bionanocomposite films loaded with different contents of rGO and ZnO-rGO was measured by two different ways, in-plane and through-plane of the film. For both measurements, Figure 5 shows that electrical conductivity values are dependent on fillers amount incorporated into alginate matrix. The highest contents of rGO and ZnO-rGO fillers allow obtaining the highest values of electrical conductivity. It is noteworthy that due to the preferential alignment of rGO sheets parallel to the plane, the electrical conductivity measured in-plane is higher in three dimensions when compared with the value through-plane [27,63]. Regarding the in-plane conductivity (Figure 6), the bionanocomposite film with 50% of ZnO-rGO reached the highest value, around 0.1 S/m, significantly different of the value obtained for the film only with rGO (0.07 S/m). In fact, ZnO particles can act as a join point enhancing the electrical conductivity. The defects that remain in the GO sheets after reduction prevent the transport of charge carriers, but a hopping mechanism can occur if ZnO is present. An alternative path for charge transport can be provided through electrostatic attraction between the Zn^2+^ ions with the negative charges of functional groups of rGO [64]. A study also supports this hypothesis when applied rGO-ZnO-MWCNT in a polymeric matrix of PVC, demonstrating that the presence of ZnO in the composite was more efficient to enhance the electrical conductivity when compared with rGO-MWCNT [65]. According to the literature, rGO introduced into a polymeric matrix composed by gelatine/sodium alginate/hyaluronic acid obtained a lower in-plane conductivity, 1.19 × 10^−3^ S/m, with 10% of filler loading [66]. Another study showed that the rGO present in alginate biopolymer increased the electrical conductivity to 10^−1^ S/m using an amount of filler beyond 12% [67], obtaining an in-plane electrical conductivity value in the same order of magnitude as the best film of this work. 

The perpendicular films’ electrical conductivity (through-plane) is not as explored in the literature, but the analysis of this parameter is essential when aiming to find the potential application for food treatment by PEF or ohmic heating. In fact, the electric field needs to pass perpendicular to the film packaging surface to reach the food product to inactivate the bacteria. Figure 5b shows that the samples with the addition of 50% of filler obtained the highest values of conductivity through-plane, about 7.5 and 11.5 × 10^−5^ S/m for the 50ZnO-rGO and 50rGO, respectively. Both samples have superior conductive values when compared with a chitosan-based film containing 50% of rGO produced by a green methodology, which achieved an electrical conductivity of 2.1 × 10^−5^ S/m [27].

### 2.5. Film Active Properties—Antioxidant and Antimicrobial Activities

The antioxidant activity of all alginate-based films was determined by the films capacity to scavenge free radicals, using the ABTS methodology. Figure 6 displays the percentage of inhibition during half an hour of film exposure to ABTS radical cation in solution. The alginate film control was able to inhibit around 6% of ABTS radical cation, while the bionanocomposite films reached an inhibition 4 and 6 times higher, with a slight increase of the values with the filler content. Moreover, there is no significant difference in antioxidant activity when comparing films at the same concentration of rGO or ZnO-rGO. The increased antioxidant activity of bionanocomposite films occurs due to the contribution of both fillers, since they were already described individually with radical scavenging activity [27,68], but also due to synergetic effects between the zinc oxide particles and the reduced graphene oxide sheets [29]. The high antioxidant activity of the bionanocomposite films imparts to these materials active properties for food packaging application, where the food product is preserved during the storage through the prevention of lipidic oxidation processes or by maintaining its original flavour and colour [69].

The antimicrobial activity of bionanocomposite films with 50% of rGO and ZnO-rGO filler was studied to evaluate their potential as active food packaging. A modified direct contact test was applied to evaluate the films’ antibacterial activity, simulating a direct contact between a solid food product and the packaging material. The films were left in contact with the TSA (trypticase soy agar) medium, previously inoculated with bacteria, for 4 h at 4 °C, temperature normally used for refrigerated food. After that, films were removed and the plate was placed at 37 °C to promote bacteria growth (Figure S2). Both microorganisms under study are facultative anaerobic, growing faster in the presence of oxygen, so removing the films from the plate ensures that aerobic growth of microorganisms is not affected. The results showed that no growth for both *E. coli* and *S. aureus* was observed in the areas where 50ZnO-rGO film was in contact with the medium. In contrast, control film (CA film) and alginate film with only rGO (50rGO film) were not effective at reducing the bacteria growth. Therefore, the presence of ZnO particles plays a crucial role in the antibacterial performance of ZnO-rGO composite filler. Despite the low diffusion of ZnO particles from the ZnO-rGO composite and from the alginate matrix to the growth medium, those present on the film surface were effective in inhibiting the bacterial growth where they were in direct and static contact with bacteria. This result demonstrates the potential interest of the application of ZnO-rGO in the alginate as an active material for food packaging.

## 3. Materials and Methods

### 3.1. Materials

Graphite (~150 µm flakes), alginic acid sodium salt (1.56 M/G), sepiolite, H_3_PO_4_ (≥85%), H_2_SO_4_ (97%), HCl (37%), KMnO_4_ (99.0%), and H_2_O_2_ (30%) were purchased from Sigma-Aldrich Co (St. Louis, MO, USA). Zinc acetate dihydrate and NH_4_OH solution were obtained from Honeywell Fluka (Charlotte, NC, USA), while NaOH was supplied by LabChem Inc (Zelienople, PA, USA). Glycerol (95%) was purchased from Scharlab, S.L. (Barcelona, Spain) and CaCl_2_ dihydrate acquired by Merck (Darmstadt, German). All other reagents were of analytical grade and used without further purification.

### 3.2. Synthesis of rGO and/or ZnO-rGO

Graphene oxide (GO) was obtained from graphite powder using an improved Hummers method [70] (experimental details in Supplementary Materials) and ZnO-rGO composite was produced according to the method described by Marlinda et al. [71]. A solution of 0.1 M Zn(OH)_2_ was prepared using 2.75 g of Zn(CH_3_COO)_2_·2H_2_O and 1 g of NaOH dissolved in 25 mL of 25% NH_3_·H_2_O. To 20 mL of GO solution (12.5 mg/mL), 2 mL of 0.1 M Zn(OH)_2_ solution was added under stirring. Then, the mixture with a pH of 12 was put at 60 ± 2 °C to obtain a dark brown solution. After 10 min, the solution was transferred to a Teflon-lined stainless-steel autoclave (50 mL) to be subjected to hydrothermal treatment at 180 °C for 24 h. After cooling to room temperature, the product was washed with water and ethanol until neutral pH. The rGO was obtained using the same procedure, except for the addition of zinc precursor to the mixture.

### 3.3. Preparation of Alginate-Based Films

ZnO-rGO particles suspension in water (25, 30, 40, and 50% wt of alginate) were dispersed with sepiolite (25% wt of alginate) using a sonication tip of 3 mm (20 min, 50% amplitude, 10 s on/off). Sodium alginate powder was then added to the ZnO-rGO/sepiolite suspensions to obtain a content of 1.5% (*w*/*v*) and dissolved overnight at room temperature with stirring. Glycerol (50% wt of alginate) was added to the mixtures and stirred for 10 min at 50 °C. The mixtures were then filtrated through a nylon mesh cloth and degassed under vacuum. Alginate-based films were obtained by solvent casting method. The dispersions (31 g) were transferred to a plexiglass plate with 144 cm^2^ and 3 mm deep and dried at 30 °C for 16 h in an air circulating oven. For comparison, an alginate control film and alginate films with rGO dispersed in sepiolite were also prepared.

All the films were immersed in a 2% CaCl_2_ aqueous solution for 3 min and, then, washed with distilled water. Afterwards, the films were immersed in a glycerol aqueous solution (75:25 of glycerol:water) at room temperature for 16 h, washed again with distilled water to remove the excess of glycerol, and dried at room temperature. The films were stored for 5 days under controlled equilibrium relative humidity (54%) at room temperature until further use. Films were named according to the type of filler and its loading, for example, films with the addition of 25% of ZnO-rGO were named 25ZnO-rGO, whereas the control alginate film (0% of ZnO-rGO and rGO) already immersed in the calcium solution was named CA.

### 3.4. Characterization of Alginate-Based Films

The structural and morphologic properties of all alginate-based films were evaluated by powder X-ray diffraction (XRD), Raman spectroscopy, and Scanning Electron Microscopy (SEM) paired with energy dispersive spectroscopy (EDS). Performance properties like water solubility, wettability, water vapour permeability (WVP), and mechanical properties (tensile strength, Young’s modulus, elongation at break) were also evaluated. Besides the measurement of electric conductivity in-plane and trough-plane the film, antioxidant activity by ABTS method and antimicrobial activity against *Escherichia coli* (ATCC25922) (Gram-negative) and *Staphylococcus aureus* (ATCC29213) (Gram-positive) were also studied to assess the active properties of the films. Full experimental details about all these characterization techniques are available in Supplementary Materials (Section 1.2). 

### 3.5. Statistical Analysis

The results of solubility, mechanical properties, surface wettability, antioxidant activity, water vapor permeability, and electrical conductivity were statistically evaluated using the analysis of variance (ANOVA) procedure in SPSS (trial version 24, SPSS Inc., Chicago, IL, USA, IL0 software). Tukey’s Honestly Significant Difference (HSD) was used at the 95% confidence level. 

## 4. Conclusions

Alginate films incorporated with rGO or ZnO-rGO fillers, previously dispersed in an aqueous suspension of sepiolite fibres, were produced by solvent casting. The complexation treatment of bionanocomposite films with Ca^2+^ was useful to maintain their integrity in a water solution. The addition of fillers into the alginate matrix originates a slight increase of WVP and wettability of bionanocomposite films. However, the interfacial adhesion between the fillers and the alginate matrix should be improved to obtain a better mechanical performance. The homogeneous dispersion of fillers allowed to increase the electrical conductivity of alginate films with a notable enhancement of the through-plane electrical conductivity values, 40 and 30 times for 50rGO and 50ZnO-rGO films comparatively to the control alginate film. This is a fundamental parameter to allow the passage of electric current through the film aiming the packaged food sterilization. Although the good electric conductive values, the 50ZnO-rGO film was shown to be more promising due to its active properties, namely its antioxidant activity (38% of ABTS inhibition in half hour) and antimicrobial activity against *E. coli* e *S. aureus*. In this context, the film’s electrical conductivity combined with the active properties leads to the formation of a very promising and suitable alginate bionanocomposite material for packaging industry, aiming the extension of food products’ shelf-life. 

## Data Availability

The data is included in the main text and/or the Supplementary Materials.

## Supplementary Materials

The following are available online at https://www.mdpi.com/article/10.3390/ijms22189943/s1.
