# Supplementary material for: Design of Alginate-Based Bionanocomposites with Electrical Conductivity for Active Food Packaging
Source: Int J Mol Sci. 2021 Sep 14;22(18):9943. doi: 10.3390/ijms22189943 (PMC8466826; doi:10.3390/ijms22189943)
Supplement: Supplementary file 1 [file ijms-22-09943-s001.zip › ijms-1360557-supplementary.pdf]

Supplementary Materials

Design of alginate-based bionanocomposites with electrical conductivity for active food packaging

Zélia Alves,[a,b] Nuno M. Ferreira,[c] Sónia Mendo,[d] Paula Ferreira,[b*] Cláudia Nunes,[a*]

[a]Department of Chemistry, CICECO - Aveiro Institute of Materials, University of Aveiro, 3810-193 Aveiro, Portugal

[b]Department of Materials and Ceramic Engineering, CICECO - Aveiro Institute of Materials, University of Aveiro, 3810-193 Aveiro, Portugal

[c]Department of Physics, i3N, University of Aveiro, 3810-193 Aveiro, Portugal

[d]Department of Biology, CESAM, University of Aveiro, 3810-193 Aveiro, Portugal

Corresponding authors:

Paula Ferreira, pcferreira@ua.pt; Cláudia Nunes, claudianunes@ua.pt

Phone:+351 234 372581

1. Experimental details

1.1. Synthesis of graphene oxide (GO)

Graphene oxide was obtained from graphite powder using an improved Hummers method. Firstly, 180 mL of H_2SO_4 and 20 mL of H_3PO_4 were added to graphite (1.5g). The $KMnO_4$ (9 g) was added slowly to the mixture under stirring, producing a slight exotherm reaction. The reaction was heated to 50 ºC and stirred for 12 h. Then, the mixture flask was cooled to room temperature and, subsequently, 200 mL of ice was poured into the mixture, followed by around 1.5 mL of H_2O_2 (30%) until the mixture colour turns yellowish. The mixture was centrifuged (6000 rpm for 30 min), and the supernatant was separated and discarded. The remaining solid material was then washed once with distilled water, followed by HCl (30%), and twice with ethanol. Finally, the material was dissolved in water and was exfoliated through ultrasonication for 45 min (SONOPLUS HD 3100, 45W, pulse 10 s and pause 5 s) [1].

1.2. Characterization of alginate-based films

1.2.1. Structural and morphologic characterization

X-ray diffraction (XRD) was performed on a Panalytical Empyrean X-ray diffractometer (Malvern Panalytical, Malvern, UK) with Cu-K_α radiation (λ =1.54178 Å). The synthetized particles and the alginate-based films were recorded in a reflection and transmission mode, respectively, with the scanning angle ranged (2θ) from 5° to 70°. The diffractograms of alginate-based films were normalized with a reference diffraction peak which is common in all samples, in this case, the 2θ = 6.14° corresponding to the (110) plane. Raman spectroscopy was carried out using Jobin Yvon T64000 instrument (HORIBA, Kyoto, Japan) equipped with a laser operating at 441 nm as an excitation source wavelength laser. The surface and cross-section morphology of the

bionanocomposite films were observed using a high-resolution scanning electron microscopy (SEM) (SU-70 Hitachi microscope, Hitachi High-Tech Corporation, Tokyo, Japan) operating at 15 kV and 15 mm of working distance. The elemental compound of films was investigated using energy dispersive spectroscopy (EDS, Bruker Quantax 400). Carbon tape was used to fix the samples on the SEM specimen holder and sputter coated with carbon.

1.2.2. Water solubility, wettability, and water vapour permeability (WVP)

The films solubility was determined according to the method described by *Nunes et al.* [2]. One square (4 cm^2) of film, previously weighted, was placed in 30 mL of distilled water (pH 6.5), containing sodium azide, at room temperature for 7 days with orbital agitation (80 rpm). Afterwards, the films were dried at 105 ºC for 16 h, cooled in a desiccator containing phosphorous pentoxide until room temperature, and reweighted. The solubility was determined by the percentage of weight loss of the film, on a dry weigh basis, where initial film weight was corrected considering the initial films moisture. The analysis was carried out with three independent assays.

Water contact angle on the surface (wettability) of films (strips of 1 × 10 cm) was performed, at room temperature, using a sessile drop of 3 μL of ultrapure water dispensed on the film surface. An OCA instrument (Dataphysics) was used for the measurements and the contact angle of the drops was calculated based on the Laplace-Young method with an image analysis software (Dataphysics SCA20M4). At least ten droplets were measured for down and up film surface, where down and up means the film surface that is contact with the plexiglass plate and the air during the solvent casting, respectively.

Water vapour permeability (WVP) was measured following a previously described method based on the ASTM standard method E 96-95 (desiccant method) [3]. Three samples of each film were tested. Films were glued to an aluminium adhesive tape, which was perforated with a

diameter of 2.2 cm, and sealed on cups containing a fixed amount of anhydrous calcium carbonated, dried at 200 °C before use. Cups were placed into controlled moisture and temperature chamber (53%, 25 °C), which is equipped with a fan to ensure homogeneity of the relative humidity. The cups were periodically weighted over time until the weight gain become constant. Water vapour transmission rate (WVTR) (g m^{-2} s^{-1}) was calculated by dividing the slope of a linear regression of weight gain versus time by film, and WVP (g m^{-1} s^{-1} Pa^{-1}) was measured applying the Equation (1)

$$WVP = \frac{(WVPR \times L)}{\Delta P} \quad (1)$$

where L is the film thickness (m) and ΔP is the water vapour partial pressure difference (Pa) between both sides of the film.

1.2.3. Mechanical properties

According to the standard method (ASTM D 882-83), tensile tests of films was performed using a texture analyzer apparatus (model TA.XT*plus*C, Stable Micro Systems, Godalming, UK) equipped with fixed grips with an initial separation of 50 mm. Films were cut in strips of 70 × 10 mm, fixed on the grips and stretched at a constant crosshead speed of 0.5 mm/s. At least six samples of each film were tested, which were previously conditioned for 5 days under controlled moisture (45%) and temperature conditions (22 °C). Mechanical properties, such as tensile strength, elongation at break, and Young's modulus, were determined from stress-strain curves. The films' thickness was measured in three different points along the strip using a digital micrometre with ± 0.001 mm accuracy (Mitutoyo Corporation, Takatsu-ku, Kawasaki, Kanagawa Japan).

1.2.4. Electrical conductivity measurements

Films' electrical conductivity was evaluated in-plane and through-plane of the film using a home-made resistivity setup prepared with 4-point probe and 2-point probe, respectively [4]. Three strips (0.5 × 3.5 cm) and three squares (1 cm^2) of each film were cut to carry out the in-plane and through-plane measurements, respectively. The electrical response was made at room temperature by direct current (*dc*) measurements using a programmable power supply IPS603 (ISO-Tech) and a Multimeter 34401A (HP). The value of electrical conductivity (S/m) for each film was then measured following the Equations (1) and (2).

$$R = \frac{V}{I} \quad (1) \qquad \sigma = \frac{L}{R \times S} \quad (2)$$

Where R is the resistance, V means tension, I is the current, σ is the electrical conductivity, L and S are the length and the cross-sectional area for each specimen.

1.2.5. Antioxidant activity – ABTS assay

A modification of ABTS method was used to evaluate films' antioxidant activity [2]. Firstly, a solution of 7 mM ABTS was prepared in 2.45 mM potassium persulfate and kept in dark at room temperature for 16 h, allowing the ABTS radical cation formation. The ABTS radical cation solution was diluted in water (1:80) and its concentration was adjusted to 0.700–0.800 absorbance values measured at 734 nm (Powerwave HT, BioTek spectrophotometer microplate reader, Winooski, Vermont, EUA). One film square (1 cm^2) was placed in 3 mL ABTS radical cation solution and left to react under dark conditions at room temperature with orbital stirring (80 rpm) over 24 h. The differences in absorbance allowed to calculate the ABTS radical cation inhibition percentage during the incubation time. ABTS radical cation solution without film was used as blank. All measurements were performed in triplicate.

1.2.6. Antimicrobial activity

The antibacterial activity of films (CA, 50rGO and 50ZnO-rGO) was evaluated against two different food-borne pathogenic bacteria, *Escherichia coli* (ATCC25922) (Gram-negative) and *Staphylococcus aureus* (ATCC29213) (Gram-positive). The bacterial pre-inoculum was prepared in TSB (tryptic soy broth) medium and incubated with shaking (180 rpm) for 16-24 h at 37 ºC. Subsequently, 100 µL of this fresh culture, at a final concentration of $10^6 - 10^7$ CFU/mL were uniformly spread over TSA (tryptic soy agar) plates. Films were cut with a diameter of 6 mm, sterilized under ultraviolet radiation for 10 min in each side, placed over the agar, and kept at 4 °C for 4 h. Then, films were removed, and the plates incubated at 37 °C for 16-24 h. Three independent assays were performed for each sample and the bacterial growth inhibition was observed in the area where the film was placed.

2. Results and Discussion

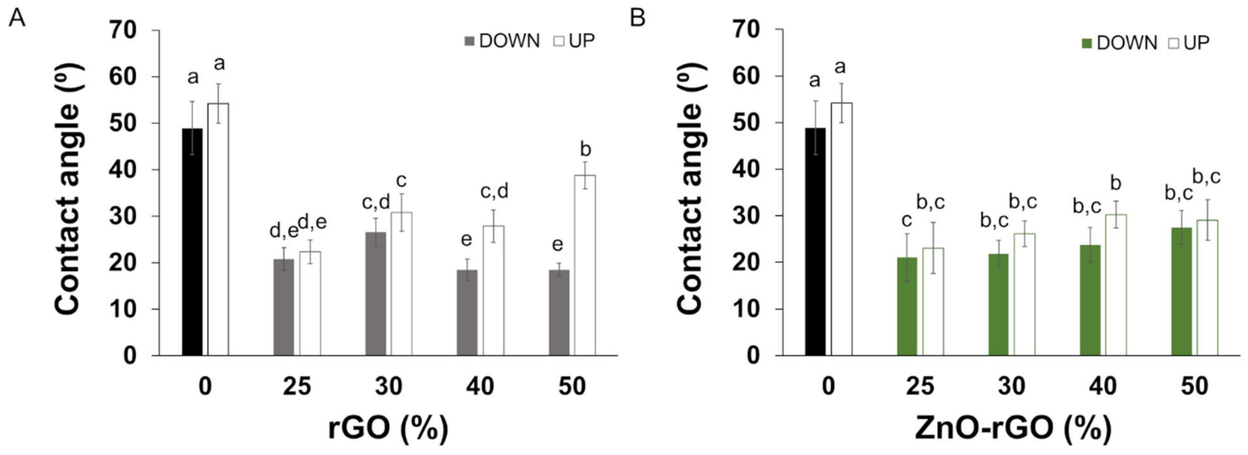

Figure S1. Water contact angle (WCA) of CA and bionanocomposite films with (A) rGO (25rGO, 30rGO, 40rGO, and 50rGO) and (B) ZnO-rGO (25ZnO-rGO, 30ZnO-rGO, 40ZnO-rGO, and 50ZnO-rGO). Different letters represent significant ($p < 0.05$) values (n=10).

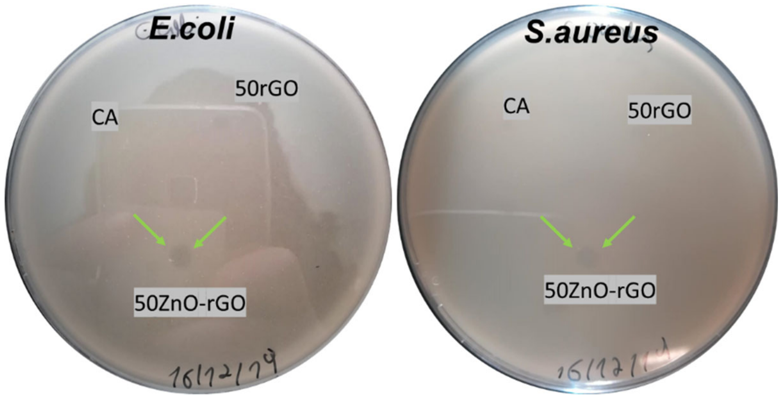

Figure S2. Antibacterial activity by a direct contact method of CA, 50 rGO and 50ZnO-rGO films against *E. coli* and *S. aureus* food-borne pathogenic bacteria. The arrows show the inhibition area where 50ZnO-rGO film was in contact.

3. References

[1] D.C. Marcano, D. V Kosynkin, J.M. Berlin, A. Sinitskii, Z. Sun, A. Slesarev, L.B. Alemany, W. Lu, J.M. Tour, Improved Synthesis of Graphene Oxide, ACS Nano. 4 (2010) 4806–4814.

[2] C. Nunes, É. Maricato, Â. Cunha, A. Nunes, J.A. Lopes, M.A. Coimbra, Chitosan – caffeic acid – genipin films presenting enhanced antioxidant activity and stability in acidic media, Carbohydr. Polym. 91 (2013) 236–243.

[3] Standard Test Methods for Water Vapor Transmission of Materials (E 96 - 95), in: Annu. B. ASTM Stand., ASTM, Philadelphia, 2002.

[4] R.K. Hiremath, M.K. Rabinal, B.G. Mulimani, Simple setup to measure electrical properties of polymeric films, Rev. Sci. Instrum. 77 (2006) 1–4.
